# A 6-year Cohort Study on Relationship between Functional Fitness and Impairment of ADL in Community-dwelling Older Persons

**DOI:** 10.2188/jea.13.142

**Published:** 2007-11-30

**Authors:** Toshiya Nagamatsu, Yukio Oida, Yoshinori Kitabatake, Hiroshi Kohno, Ken’ichi Egawa, Naomi Nezu, Takashi Arao

**Affiliations:** 1 Physical Fitness Research Institute, Meiji Life Foundation of Health and Welfare.; 2 Health Department, City of Enzan.

**Keywords:** cohort studies, functional fitness, ADL impairment, community-dwelling aged persons

## Abstract

We measured functional fitness in older subjects and performed a follow-up survey for 6 years to clarify whether the level of functional fitness at a given point contributes to prediction of the subsequent occurrence of impairment of functions necessary for independent living. The longitudinal data were obtained for 391 persons aged 60 years or over, who were independently living in the community. Four items of functional fitness, i.e. the ability to perform standing/sitting movements, ability to perform traveling movements, ability to perform housekeeping movements, and ability to perform personal grooming activities, were assessed. The relationship between functional fitness and the risk of the occurrence of impairment of independence in daily living was evaluated using a binomial logistic regression model. In males, all the 4 items of functional fitness were significantly related to the risk of impairment of activities of daily living (ADL). In females, however, none of the items was significantly related to the risk of ADL impairment. In conclusion, functional fitness appears to be valid as a predictive parameter of future occurrence of ADL impairment in relatively healthy and independent elderly males. However, its validity in elderly females needs further evaluation.

In Japan, where the population is rapidly aging, not only increasing the life span by preventing diseases but also prolonging active life expectancy with satisfactory quality of life (QOL) have become important issues. In such social circumstances, study of the relationship between the state of physical functions necessary for independent living and the future outcome, such as the incidence of impairment of activities of daily living (ADL) or mortality, in the general elderly population may provide useful information for planning and implementing health promoting services for prolonging the active life span of older people.

In Japan, there have been a number of studies examining the relationship between physical functions necessary for ADL and various factors in daily living in groups that included elderly people with impaired physical functions or disabilities.^1^^-^^4^ However, few studies have objectively assessed physical functions necessary for ADL and examined their relationships with other factors or the outcome in independently living elderly people. We defined physical abilities necessary for the elderly to live an independent life as “functional fitness” and have developed a method for their objective measurement.^5^^-^^10^ We have carried out cross-sectional studies of elderly people and clarified the relationships among functional fitness with the state of medical health, state of daily living, and health behavior.^11^ However, whether the level of functional fitness allows prediction of the outcome, such as future impairment of ADL or death, has not been sufficiently evaluated. Evaluation of the relationship between functional fitness and the outcome will clarify the significance of measurement and assessment of functional fitness, and provide basic data useful for planning health-promoting programs and organizing specific actions of community care aimed at maintenance and enhancement of these functions and prevention of reductions in the degree of independence in daily living or total disability.

In this study, we measured functional fitness in elderly subjects and performed a follow-up survey for 6 years to clarify whether the level of functional fitness at a given point contributes to prediction of the subsequent occurrence of impairment of functions necessary for independent living.

## METHODS

### Subjects

The subjects were 397 volunteers (160 males, 237 females) aged 60 years and older who lived independently at home in Enzan city located in the northeast part of Yamanashi Prefecture, Japan. Enzan city had a population of 27,126, and the proportion of the population aged 65 years and older was 18.1% in 1992. The baseline survey was carried out between November 1992 and January 1993. The follow-up survey was conducted between December 1998 and March 1999. Subjects who lived far from the investigation sites were picked up at home and returned by car. Prior to the investigation, all the subjects were informed about the purpose and contents of the investigations, and their written consent to enrollment was obtained. These investigations were carried out as “Health Measurement and Monitoring Service for the aged persons” sponsored by the public health promotion center in the city, with the cooperation of the senior citizens’ association. Of the 397 subjects enrolled in the baseline investigation, 366 (148 males, 218 females) in whom the values of all the 4 evaluation items of functional fitness (standing /sitting ability, walking ability, hand working ability, and self-care working ability) were available and in whom ADL could be evaluated at the follow-up investigation were included in the analyses. Thirty one subjects who could not complete the items at baseline, refused, or were absent, or out of town at follow-up were excluded from the subjects for analysis. The follow-up rate was 92.2%. Table 1 shows the age and sex distribution of the subjects included in the analyses and the mean and standard deviation of the 4 items of functional fitness. The means and standard deviations of age for males and females were 76.7 ± 5.7 years and 75.4 ± 5.7 years, respectively.

**Table 1.** Age and sex distribution of the subjects included in the analyses and the mean and standard deviation of the 4 items of functional fitness at baseline.

Age (year)	n	Standing/sitting (sec)	Walking (sec)	Hand working (sec)	Self-care working (sec)
male					
-64	1	5.0 (3)	6.1 (4)	32.0 (4)	5.8 (4)
65-69	17	5.4 ± 1.2 (3)	7.3 ± 1.0 (4)	34.4 ± 5.9 (4)	7.3 ± 1.6 (2)
70-74	39	5.3 ± 1.1 (4)	7.3 ± 1.4 (3)	34.4 ± 3.5 (4)	7.4 ± 1.5 (3)
75-79	44	7.1 ± 3.7 (3)	8.3 ± 1.9 (3)	38.4 ± 8.1 (4)	8.8 ± 3.4 (2)
80-84	32	6.9 ± 2.0 (3)	8.6 ± 1.6 (3)	36.8 ± 3.8 (4)	8.8 ± 3.1 (3)
85-89	14	7.7 ± 1.2 (4)	9.1 ± 1.5 (3)	39.6 ± 5.2 (4)	9.0 ± 2.0 (4)
90+	1	9.7	7.8	45.0	7.8
Total	148				

female					
-64	6	4.7 ± 1.1 (4)	7.1 ± 0.6 (3)	28.3 ± 2.2 (5)	6.7 ± 0.8 (2)
65-69	29	5.7 ± 1.6 (4)	7.6 ± 1.0 (3)	32.1 ± 3.0 (4)	7.3 ± 1.5 (2)
70-74	61	6.7 ± 1.7 (3)	8.4 ± 1.6 (3)	33.5 ± 3.6 (5)	7.7 ± 1.4 (2)
75-79	73	7.8 ± 2.8 (3)	8.9 ± 1.4 (4)	35.9 ± 4.3 (4)	8.4 ± 2.2 (2)
80-84	38	9.9 ± 4.6 (3)	9.8 ± 2.1 (3)	37.9 ± 4.1 (5)	9.4 ± 2.7 (2)
85-89	10	11.0 ± 4.3 (3)	11.7 ± 7.2 (4)	39.0 ± 5.1 (5)	9.6 ± 2.0 (2)
90+	1	8.5	9.1	39.0	11.2
Total	218				

Mean ± standard deviation.

Evaluated score according to standard value of functional fitness in parentheses ( 1 : poor → 5 : good ).

### Contents of investigations

Height, body weight, functional fitness, and the degree of independence in ADL were measured at 8 investigation sites in the city. Body mass index (BMI) was calculated by height and body weight.

### Inquiry

Inquiry by physicians and nurses was made before the measurement of functional fitness. The subjects were asked whether they had diseases of the cardiovascular and musculo-skeletal systems, their medical history, and whether they had pain in the upper or lower limbs or lower back. A history of symptoms and current symptoms were regarded as positive when the subject was diagnosed and treated by a physician in the past or at the time of the investigation. Concerning pain of the upper and lower limbs and lower back, “symptoms of musculo-skeletal disorders” were considered to be positive when the subject who always or sometimes had pain, reported pain on the day of the investigation. At the baseline investigation, degree of independence in daily living was evaluated. At the follow-up investigation, confirmation of the death or degree of independence in daily living was evaluated.

### Functional fitness

Four items of functional fitness, i.e. the ability to perform standing and sitting movements (standing/sitting ability), ability to perform traveling movements (walking ability), ability to perform housekeeping movements (hand working ability), and ability to perform personal grooming activities such as dressing, bathing, and applying make-up (self-care working ability), were assessed. The standing/sitting ability was evaluated by measuring the time required to complete a series of movements (going from a supine to a standing position, sitting on a chair and then standing up again). The walking ability was evaluated by measuring the time taken to walk alone a 10 meter distance with 4 zigzags (two turns to the right and two to the left, around markers on the floor 50 cm distant from the central line, placed at intervals of 2 meter). The hand working ability was evaluated using a “peg board” test authorized by the Japanese Ministry of Health, Labor and Welfare as an occupational aptitude test. The examinee transferred two pegs at a time from one set of holes to another set, using both hands. The time required for the examinee to transfer all the 48 pegs to other holes was measured. The self-care working ability was evaluated by measuring the time taken to perform a sequence of 3 test movements. The test sequence in this case was to grip both ends of a rope equivalent in length to the distance from the fingertip of one horizontally extended arm to the acromion of the other shoulder. Then the participant stepped across the rope, on leg after the other, while standing. Finally, the participant lifted the rope behind the back and over the head, back to the initial standing position with the rope in front of his or her body. The participant was instructed to attempt to complete each item in the sequence as quickly as possible. Each item was examined twice after a single practice, and the better result was accepted. Each of the 4 items of functional fitness was expressed as the Z score using the mean and standard deviation for males and females separately, the scores for the 4 items were summed, and the total Z score of functional fitness (T-Z score) was calculated by multiplying the sum by −1 as a parameter for comprehensive evaluation of functional fitness.^12^

### Degree of independence of daily living

The degree of independence in daily living was assessed in the survivors using the “Evaluation Criteria for the Degree of Independence in Daily Living (Bed-Ridden Scale) for the Disabled Elderly” by the Ministry of Health, Labor and Welfare.^13^ The investigation was carried out by direct interviews or telephone interviews of the subjects or their families by trained public health nurses. Subjects at rank J were evaluated to be independent in daily living. Subjects at rank A, B, or C were evaluated to be house-bound, chair-bound, or bed-bound respectively. In this study, death was regarded as the terminal stage of health disruption, and being on a care-dependent level as the stage previous to the final stage of health disruption. Therefore, subjects who died and ranked A, B, or C in bed Ridden Scale (care-dependent individuals) were defined as individuals having impairment of ADL (ADL-impaired individuals).

### Statistical analyses

The relationship between functional fitness and the risk of the occurrence of impairment of independence in daily living (risk of ADL impairment) was evaluated using logistic regression models. In these analyses, 1 was given to individuals with ADL impairment, and 0 was given to independent individuals (rank J) as dummy variables, and re-coded values were used as the dependent variable. The age at the baseline investigation, the history or current presence of cardiovascular disorders (positive: 1; negative: 0), the history or current presence of locomotive disorders (positive: 1; negative: 0), and BMI were used as covariate variables, and the odds ratios and their 95% confidence intervals were calculated for males and females separately. The analyses were carried out using SPSS^®^ 10.0J software for Windows^®^, and the results were considered significant when the 95% confidence interval did not include 1.0.

## RESULTS

### Characteristics of subjects at baseline investigation

The degree of independence in all our subjects at the baseline investigation was rank J.

### Distribution of health state after 6 years

Figure summarizes the outline of sampling for analysis. During the 6 years, 59 subjects (30 males and 29 females) died, and the cumulative mortality rate during this period was 15.1%. Of the 307 survivors (118 males and 189 females) after exclusion of 25 subjects (11 males and 14 females; 6.4% of all subjects) who could not be followed up because of loss or rejection of investigation, 286 (111 males and 175 females) were independent, and 21 (7 males and 14 females) were care-dependent.

**Figure.**
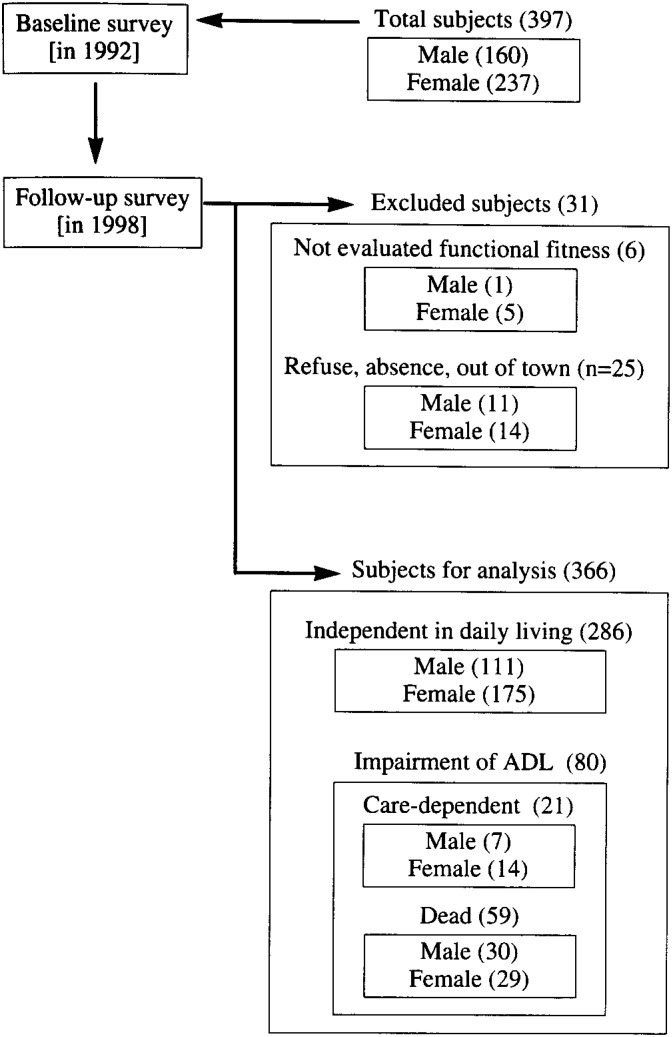
Outline of sampling for analysis in the study. Number of subjects in parentheses.

Table 2 shows the state of the occurrence of ADL impairment in the subjects included in the analyses by age and sex. The cumulative incidence of ADL impairment during the 6 years tended to increase with the age of the subjects.

**Table 2.** Occurrence of ADL impairment in the subjects included in the analyses by age and sex.

Age at baseline (year)		Male			Female		
	
		Care-dependent	Dead	Total	Care-dependent	Dead	Total
60-69	(18 males and 35 females)	0	0	0	0	3	3
70-79	(83 males and 134 females)	3	20	23	10	15	25
80+	(47 males and 49 females)	4	10	14	4	11	15

Total	(148 males and 218 females)	7	30	37	14	29	43

Care-dependent: Rank A, B, or C in Bed-Ridden Scale

### Functional fitness and odds ratios of the occurrence of ADL impairment

Table 3 shows odds ratios of the occurrence of ADL impairment for the T-Z score of functional fitness and measured items. In the males, the standing/sitting ability, walking, hand-working ability, self-care working ability, and T-Z score were all significantly related to the risk for ADL impairment. When the correlations among these 4 items were examined with adjustment for age, the partial correlation coefficients ranged from 0.477 to 0.639, and were significant (p<0.01). In the females, however, none of the items were significantly related to the risk for ADL impairment. The scores of the 4 evaluation items of functional fitness in the males were categorized into 5 levels (by 20 percentile), and the odds ratios of impairment of items were calculated for the second, third, fourth, and fifth (lowest) categories compared with the highest (best) 20 percentile (Table 4). In the males, the odds ratio was significantly greater in the lowest group than in the highest group for the times assessed in standing/sitting ability, walking ability, hand working ability, and self-care working ability. The odds ratio increased significantly when the time assessed in standing/sitting ability was greater than 7.5 seconds, the time assessed in walking ability was greater than 9.1 seconds, the time assessed in hand working was greater than 40.0 seconds, and the time assessed in self-care working was greater than 9.2 seconds.

**Table 3.** Odds ratios of the occurrence of ADL impairment after 6 years for T-Z score and measured items.

Item	male	female
T-Z score	0.78 (0.66-0.92)	0.91 (0.81-1.02)
Standing/sitting	1.31 (1.05-1.64)	1.08 (0.97-1.21)
Walking	1.57 (1.19-2.08)	1.13 (0.96-1.33)
Hand working	1.11 (1.02-1.20)	1.03 (0.94-1.12)
Self-care working	1.23 (1.03-1.46)	1.10 (0.94-1.30)

Adjusted for age at the baseline, histories or presence of cardiovascular disorders and musculo-skeletal disorders, and body mass index.

95% confidence intervals in parentheses.

**Table 4.** Odds ratios of the occurrence of ADL impairment after 6 years of evaluation item calculated for quintile categories in males.

	Standing/sitting		Walking		Hand working		Self-care working	
			
	category	odds ratio	category	odds ratio	category	odds ratio	category	odds ratio
highest	-4.7 sec	1.00	-6.8 sec	1.00	-31.9 sec	1.00	-6.5 sec	1.00
	4.8-5.5	1.26 (0.28-5.60)	6.9-7.5	1.38 (0.30-0.65)	32.0-34.4	1.25 (0.22-7.19)	6.6-7.1	2.11 (0.44-10.1)
	5.6-6.2	1.71 (0.41-7.09)	7.6-7.9	1.35 (0.31-5.79)	34.5-36.9	4.24 (0.97-18.5)	7.2-8.2	1.41 (0.28-7.22)
	6.3-7.4	1.77 (0.41-7.63)	8.0-9.0	1.89 (0.44-8.22)	37.0-39.9	2.86 (0.61-13.4)	8.3-9.1	2.90 (0.59-14.1)
lowest	7.5+	4.88 (1.12-21.2)	9.1+	5.65 (1.28-24.9)	40.0+	5.29 (1.13-24.8)	9.2+	5.36 (1.24-23.2)

Adjusted for age at the baseline, histories or presence of cardiovascular disorders and musculo-skeletal disorders, and body mass index.

95% confidence intervals in parentheses.

## DISCUSSION

Many preceding studies have found that low level of ADL is related to a high mortality rate in the elderly, and a close association between impairment of ADL and survival has been demonstrated.^14^^-^^16^ A report also exists that defined death as the final stage of health disruption and evaluated factors related to survival.^17^ Therefore, it appears possible to understand death as the terminal stage of health disruption, the care-dependent level as the stage previous to the terminal stage of health disruption, and death and care-dependent level combined as ADL impairment. From this viewpoint, individuals who died or ranked care-dependent level were defined as ADL-impaired subjects in this study. There are a relatively large number of longitudinal studies about the relationship between survival and lifestyle that followed up groups of people aged 60 years and older. According to the 5-year follow-up study by Branch et al.,^18^ smoking was related to the state of survival in females, but no lifestyle factor screened affected the survival state in males. In the follow-up study over about 10 years by Davis et al.,^19^ a low physical activity level and a sub-average body weight were shown to be related to a short survival period in both males and females. A 17-year follow-up by Kaplan et al.^20^ showed that smoking, lack of exercise, deviation from the standard body weight, and irregular breakfast intake were related to death during the observation period. In Japan, however, few preceding studies have approached the relationship of independence in physical activities or the ability to perform physical activities with the occurrence of ADL impairment.^21^

In this study, we used functional fitness as a parameter for the assessment of ability to maintain independence in physical activities of daily living. This parameter was developed for quantitative and objective assessment of the main activities in daily living. It has also been reported that functional fitness has validity for evaluating the physical capacity of aged people.^22^ Therefore, a high score in this parameter indicates a high level of general physical functions necessary for independent living. When the 4 items of functional fitness were compared with the age and sex matched standard values,^23^ the results in our subjects were nearly average for their age or better except for the self-care working time in the females. The current study evaluated the relationship between functional fitness and the occurrence of ADL impairment after 6 years in independent local elderly people. The results obtained in this study are expected to be useful for planning and implementing health-promotion programs for prevention of ADL impairment, prolongation of the active life span, and prevention of total disability for elderly people living in the community, who account for a majority of the elderly population.

In males, the logistic analyses revealed the ability to perform standing/sitting movements, ability to perform traveling movements, ability to perform housekeeping movements, ability to perform self-care work, and the T-Z score as factors related to the risk for ADL impairment. The significant negative relationship between the T-Z score and the risk for ADL impairment indicates that maintaining overall abilities of physical activities is important for the elderly to continue leading independent lives. Sugiura et al.^21^ reported that the speed of walking decreases progressively with aging and that the maximum speed of walking is related to future death and declines in ADL. Our results were not only in agreement with those of the above earlier studies but also showed that the levels of the ability to perform standing/sitting movements, ability to perform housekeeping movements, and ability to perform self-care work were significantly related to the future occurrence of ADL impairment. Therefore, for the elderly to maintain independence in living, it is extremely important to maintain the ability to perform standing/sitting movements, housekeeping movements, and perform self-care work. However, as the correlations adjusted for age among these 4 items were significant, individual items are not considered to be independent of one another but to be mutually related by some common factors. Thus, these abilities to perform major activities of daily living are all constituted of multiple physiologic functions and elements of fitness and are equally subject to effects of senescence. Therefore, the relationships among these 4 items may reflect senescent characteristics of physical functions as a whole.

In implementing health-promoting programs for prevention of the decline of physical functions necessary for independent living and total disability, values of the test items at the point when significant odds ratio was confirmed may be useful. More specifically, implementing health-promoting activities with provisional goals of maintaining the time assessed standing/sitting ability within 7.5 seconds, the time assessed walking ability within 9.1 seconds, the time assessed hand working ability within 40.0 seconds, and the time assessed self-care working ability within 9.2 seconds is recommended. From these observations, functional fitness, which we have proposed, appears to be a valid parameter for estimation of the future risk of ADL impairment in elderly males. Preceding studies have suggested that lifestyle,^20^ physical activity level,^21^ and ADL^17^ may allow prediction of the outcome. Since diverse factors may be involved in a complex manner in the development of ADL impairment, further evaluation of non-physical factors that affect functional fitness and the occurrence of ADL impairment in elderly males may be needed.

In females, the findings were similar to those in males, but none of the items was significantly related to the occurrence of ADL impairment. This result seems to suggest that elderly females might have different physical risk factors for ADL impairment from functional fitness in aged males. It has been reported that pain(s) in the lower back and/or the knee joint was observed more frequently in aged females than in aged males,^24^ and that this pain associated with musculo-skeletal diseases was a significant risk factor of ADL impairment in the elderly.^25^ Although we adjusted for musculo-skeletal diseases at the baseline in the analysis of the relationship between ADL impairment occurrence and functional fitness level, we could not adjust for the occurrence of pain associated with musculo-skeletal diseases during the study period for the lack of the information on this event. Therefore, there might be many female subjects in whom pain appeared during the study period, and that was the reason we could not find a significant relationship between ADL impairment and functional fitness level in aged females. On this point, more detailed examination based on the information about changes in physical conditions during the study period is needed for elderly females.

From the abovementioned results, functional fitness appears to be valid as a predictive parameter of future occurrence of ADL impairment in the male elderly. However, its validity in the female elderly needs further evaluation. In addition, the subjects of this study were volunteers who belonged to a senior citizens’ association and responded to invitations to the investigation sponsored by the public health promotion center, and were not selected at random. Therefore, many of the subjects may have had a more positive lifestyle than the average elderly population. Furthermore, the measured values of the 4 items of functional fitness were better than the age and sex adjusted averages, except for the time assessed in self-care working ability in females. Thus, our subjects are likely to have been a group of individuals with relatively high levels of health-related behavior and physical functions necessary for living. For these reasons, it would be difficult to apply our findings to the local elderly in general. However, our group may adequately represent participants in health promoting programs for relatively healthy and independent local elderly people. Our results may, thus, be applicable to prospective subjects of such health promoting programs in general.
